# Impaired serial ordering in nondemented patients with mild Parkinson’s disease

**DOI:** 10.1371/journal.pone.0197489

**Published:** 2018-05-16

**Authors:** Jinghong Ma, Shaoyang Ma, Haiqiang Zou, Yizhi Zhang, Piu Chan, Zheng Ye

**Affiliations:** 1 Department of Neurology, Xuanwu Hospital of Capital Medical University, Beijing, China; 2 CAS Key Laboratory of Mental Healthy, Institute of Psychology, Chinese Academy of Sciences, Beijing, China; 3 Department of Psychology, University of Chinese Academy of Sciences, Beijing, China; 4 Department of Neurology, General Hospital of Guangzhou Military Command of PLA, Guangzhou, Guangdong, China; 5 Department of Neurology, The Second Hospital of Jilin University, Changchun, Jilin, China; 6 CAS Center for Excellence in Brain Science and Intelligence Technology, Shanghai, China; Nathan S Kline Institute, UNITED STATES

## Abstract

The ability to arrange thoughts and actions in an appropriate serial order (the problem of serial order) is essential to complex behaviors such as language, reasoning and cognitive planning. Patients with Parkinson’s disease (PD) perform poorly in tasks that rely on the successful rearrangement of working memory representations. We hypothesized that serial ordering is impaired in nondemented patients with mild PD. We recruited 49 patients with mild idiopathic PD (Hoehn and Yahr Scale 1–2.5) and 51 matched healthy adults. Nineteen patients had normal global cognition (PD-NC, Montreal Cognitive Assessment, MoCA≥26/30) and thirty patients had mild cognitive impairment (PD-MCI, 21≤MoCA≤25). All participants underwent three working memory assessments: two experimental tests that require reordering random digits following a particular rule (adaptive digit ordering test and digit span backward test) and a control test that requires maintaining but no reordering (digit span forward test). PD-NC and PD-MCI patients performed significantly worse (with lower test scores and larger ordering costs) than healthy controls in both digit ordering and backward tests, although they performed normally in the forward test. The ordering cost increased as a function of age across groups, indicating an aging-related decline in the ability of serial ordering. However, individual patients’ task performances were not correlated with their severity or duration of motor symptoms, or daily exposure to dopaminergic drugs. These results suggested that serial ordering deficits exist in early stages of PD, prior to subtle changes in global cognition and in parallel with motor symptoms.

## Introduction

In humans, the ability to arrange thoughts and actions in an appropriate serial order is essential to complex behaviors such as language production (e.g. which information to convey first) and cognitive planning (e.g. which subgoal to achieve first). The problem of serial order in behavior has been one of the most complex and far-reaching problems in psychology [1]. Patients with Parkinson’s disease (PD) perform poorly in laboratory and ecological tasks that rely on the successful rearrangement of working memory representations. For example, medicated PD patients had difficulties in finding the temporal order of letters which were presented in loop [2] or that of public events which were presented in a scrambled order [3]. They often failed to understand the temporal relation of events which were expressed out of chronological order [4, 5] or to organize a series of temporally related but randomly presented events to tell a sensible story [6, 7]. Similar ordering deficits have also been observed in unmedicated PD patients [8, 9]. In particular, PD's difficulties in rearranging events along the time axis have been linked with aberrant activations over the dorsolateral prefrontal cortex and striatum [4, 7].

Previous studies on serial ordering often pooled PD patients at early and advanced stages [6, 10] or screened dementia with less sensitive instruments (e.g. Mini-Mental State Examination) [5, 7], leaving it unclear how the impairment of serial ordering develops in PD, i.e. whether the impairment of serial ordering is a simple reflection of severe motor symptoms or global cognitive declines. We hypothesized that deficits in serial ordering exist in early stages of PD, prior to subtle changes in global cognition and in parallel with motor symptoms. To test this hypothesis, we examined behavioral performance of serial ordering tests in PD patients with mild motor symptoms (Hoehn and Yahr Scale, HY, 1–2.5) and with normal global cognition (Montreal Cognitive Assessment, MoCA≥26/30) or mild cognitive impairment (21≤MoCA≤25).

Individuals’ ability to flexibly arrange representations in verbal working memory was assessed using the adaptive digit ordering test (DOT-A) developed by Werheid et al. [10]. In this test, participants are presented three to eight digits in random order and asked to recall the digits immediately in ascending order. This test resembles classic digit span tests in that: (a) it is adaptive regarding the length of each span and discontinuation after failure within both trials of a particular span; (b) it is presented at a standard speed of one digit per second. In its requirement, crucially, the DOT-A goes beyond the simple maintenance of sequential digits to dynamically reorder the digits following a particular rule.

We additionally employed the digit span tests to rule out two alternative possibilities. First, to rule out the possibility that PD patients are unable to encode or hold original sequences, we included the digit span forward test which assesses the maintenance of sequential digits in working memory. Second, to rule out the possibility that PD patients only have difficulties in applying one specific ordering rule ('ascending'), we included the digit span backward test which also requires reordering but following a different rule ('reversing'). We predicted that nondemented PD patients show worse performance than healthy controls in both digit ordering and backward tests, but not in the digit forward test. In other words, we expected that PD patients are spared in maintaining verbal representations but impaired in rearranging the representations regardless of the ordering rule, during early stages of the disease.

## Materials and methods

All procedures were approved by the ethical committee of Xuanwu Hospital, in accordance with the Declaration of Helsinki. Written Informed consents were obtained from all participants before the experiment.

### Patients and clinical assessments

We screened 94 patients with idiopathic PD (MDS Clinical Diagnostic Criteria for Parkinson’s Disease [11]) at the Xuanwu Hospital Department of Neurology between August 2016 and April 2017. Inclusion criteria were 1) HY 1–2.5; 2) age 50–80 years; 3) education >6 years; 4) Mandarin Chinese speaking. Exclusion criteria were 1) a history of epilepsy, stroke or brain injury; 2) possible dementia (MoCA<21/30); 3) clinically significant current depression (based on patients’ medical records and current diagnosis). Forty-nine PD patients who met the inclusion and exclusion criteria were included in the analysis.

All patients were assessed on their regular antiparkinsonian drugs, including levodopa, pramipexole, piribedil, amantadine and entacapone. In addition to the levodopa actual dose, we calculated the levodopa equivalent dose to facilitate the analysis of drug effects. The former indicated the real amount of levodopa a patient took per day. The latter was the amount of levodopa that had a similar effect as all antiparkinsonian drugs taken, calculated by the formula of Tomlinson et al. [12].

Severity of motor symptoms was evaluated with the Movement Disorder Society-Sponsored Revision of the Unified Parkinson’s Disease Rating Scale (MDS-UPDRS) III (motor subscale). PD patients were grouped according to their education-adjusted MoCA (i.e. Level I assessment for diagnosing MCI in PD [13]): a subgroup of 19 patients with normal global cognition (PD-NC, MoCA≥26/30) and another subgroup of 30 patients with MCI (PD-MCI, 21≤MoCA≤25). The cutoff points of MoCA were selected following Dalrymple-Alford et al. [14].

### Healthy control subjects

We screened 94 age- and education-matched healthy adults who reported no history of significant neurologic or psychiatric disorders. Exclusion criteria were 1) possible dementia or mild cognitive impairment (MoCA<26/30); 2) possible current depression (Beck Depression Inventory II >7); 3) use of addictive drugs (e.g. opioid drugs). Fifty-one healthy controls were included in the analysis.

### Working memory tests

All participants underwent three working memory tests in a same session, including the DOT-A, digit span forward and backward tests. The three tests were identical in that a sequence of random digits was presented in each trial, at speed of one digit per second. Participants were asked to immediately recall the digits in original order in the digit span forward test, in reversed order in the digit span backward test, and in ascending order in the DOT-A.

### Statistical analysis

Data were processed with IBM SPSS Statistics 21. We first examined whether serial ordering was impaired in PD patients using repeated measures ANOVAs on raw test scores and ordering costs. The ordering cost was an index of individuals’ ability of serial ordering, calculated as score difference between the forward test and DOT-A/backward test. This contrast was to isolate the performance of digit ordering, while controlling for the performance of digit maintenance [10]. The ANOVA on raw test scores had a within-subject factor Test (DOT-A, forward, backward) and a between-subject factor Group (PD-NC, PD-MCI, healthy control). The ANOVA on ordering costs had a within-subject factor Test (DOT-A, backward) and a between-subject factor Group. Age and Education were included as covariates in both ANOVAs. To better understand the nature of interaction, the interaction between Test and Group was followed by two-sample *t* tests (two-tailed), and the interaction between Test and Age or Education was followed by partial correlation tests (two-tailed).

We then examined the effects of motor symptoms and dopaminergic drugs on serial ordering. Individual patients’ raw test scores and ordering costs were correlated with their severity of motor symptoms (MDS-UPDRS III motor score), duration of motor symptoms, levodopa equivalent daily dose or actual daily dose. Age and Education were controlled. To control for alpha error inflation in a total of twenty correlation analyses, we applied Bonferroni correction and considered significance at *p*<0.0025.

## Results

Table 1 showed demographic and clinical features of the patients and healthy controls.

**Table 1:** Demographic and clinical features of PD patients and healthy controls.

Features	PD		Healthy controls (N = 51)	Effects of group (corrected *p*)^a^
	NC (N = 19)	MCI (N = 30)		
Female: Male	11:8	15:15	27:24	n.s.
Age (years)	62.6 (6.9)	64.8 (8.5)	64.0 (5.3)	n.s.
Education (years)	13.3 (2.3)	12.4 (2.9)	12.9 (2.2)	n.s.
MoCA	27.9 (1.2)	23.6 (1.2)	28.1 (1.3)	*p*<0.01
Depression	0 (0%)	0 (0%)	0 (0%)	n.s.
Duration of motor symptoms (years)	3.7 (2.6)	3.9 (3.4)	-	n.s.
Hoehn and Yahr Scale	1.7 (0.5)	1.8 (0.4)	-	n.s.
MDS-UPDRS III Motor	26.7 (7.3)	26.7 (15.3)	-	n.s.
Levodopa actual dose (mg/day)	345.9 (252.7)	333.2 (204.2)	-	n.s.
Levodopa equivalent dose (mg/day)	451.7 (285.5)	444.7 (240.8)	-	n.s.

Mean and standard deviations, or counts and percentages, as appropriate. PD, Parkinson’s disease; NC, normal global cognition; MCI, mild cognitive impairment; MoCA, Montreal Cognitive Assessment; MDS-UPDRS, Movement Disorder Society-Sponsored Revision of the Unified Parkinson’s Disease Rating Scale.

^a^Bonferroni-corrected *p* values of one-way ANOVAs or Kruskal-Wallis one-way ANOVAs as appropriate; n.s., not significant.

**Fig 1:**
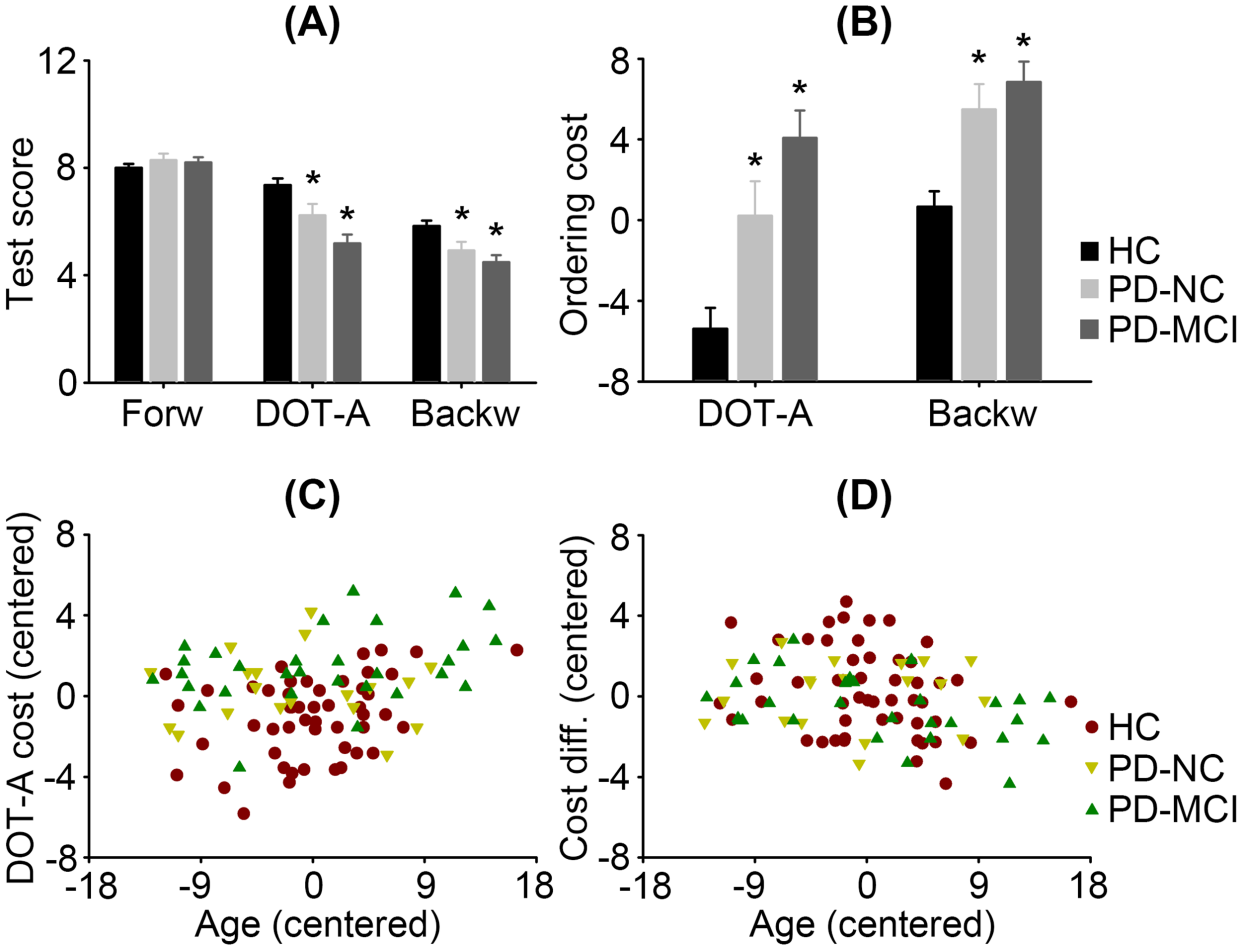
Test scores and ordering costs. (A) Mean test scores and standard errors of the adaptive digit ordering test (DOT-A), digit span forward (Forw) and backward tests (Backw) in mild PD patients with normal global cognition (PD-NC) or with mild cognitive impairment (PD-MCI) and in healthy controls (HC). The mean values were adjusted to 63.9 years of age and 12.8 years of education. Asterisks (*) indicate significant differences between patients and controls (*p*<0.05). (B) Mean ordering costs and standard errors of the DOT-A and backward test in each group. The mean values were age- and education-adjusted. Asterisks indicate significant differences between patients and controls. (C) The DOT-A ordering cost increased in older adults across groups. The values were mean-corrected. (D) Difference between the DOT-A ordering cost and backward ordering cost decreased in older adults across groups. The values were mean-corrected.

Fig 1A and 1B showed raw test scores and ordering costs, respectively, in PD-NC and PD-MCI patients as well as in healthy controls. The ANOVA on raw test scores revealed a significant interaction between Test and Group (F = 9.89, *p*<0.001) and a significant interaction between Test and Age (F = 5.60, *p* = 0.006), in addition to main effects of Test (F = 4.72, *p* = 0.013) and Group (F = 10.56, *p*<0.001). Following the Test-Group interaction, post-hoc two-sample *t* tests showed that both PD-NC and PD-MCI patients scored lower than healthy controls in the DOT-A (PD-NC: *t* = 2.00, *p* = 0.049; PD-MCI: *t* = 5.16, *p*<0.001) and backward test (PD-NC: *t* = 2.75, *p* = 0.008; PD-MCI: *t* = 4.57, *p*<0.001), but not in the forward test (*p*s>0.28). PD-NC patients scored higher than PD-MCI patients in the DOT-A (*t* = 2.50, *p* = 0.016). It means serial ordering was impaired in PD-NC patients regardless of the ordering rule and became worse in PD-MCI patients. In contrast, maintenance was preserved in those patients. Following the Test-Age interaction, post-hoc correlation tests showed that the DOT-A ordering cost, but not the backward ordering cost, increased in older adults across groups (r = 0.26, *p* = 0.009, see Fig 1C). No effect of Education was obtained.

The ANOVA on ordering costs confirmed the analysis of raw test scores. There was a significant main effect of Group (F = 21.73, *p*<0.001), indicating that both PD-NC and PD-MCI patients had larger ordering costs than healthy controls in either DOT-A or backward test. The ANOVA also revealed a main effect of Test (F = 6.46, *p* = 0.013) and a significant interaction between Test and Age (F = 5.74, *p* = 0.019). It means the backward ordering cost was larger than the DOT-A ordering cost, but their difference decreased in older adults across groups (r = -0.25, *p* = 0.014, see Fig 1D). No effect of Education was obtained.

**Fig 2:**
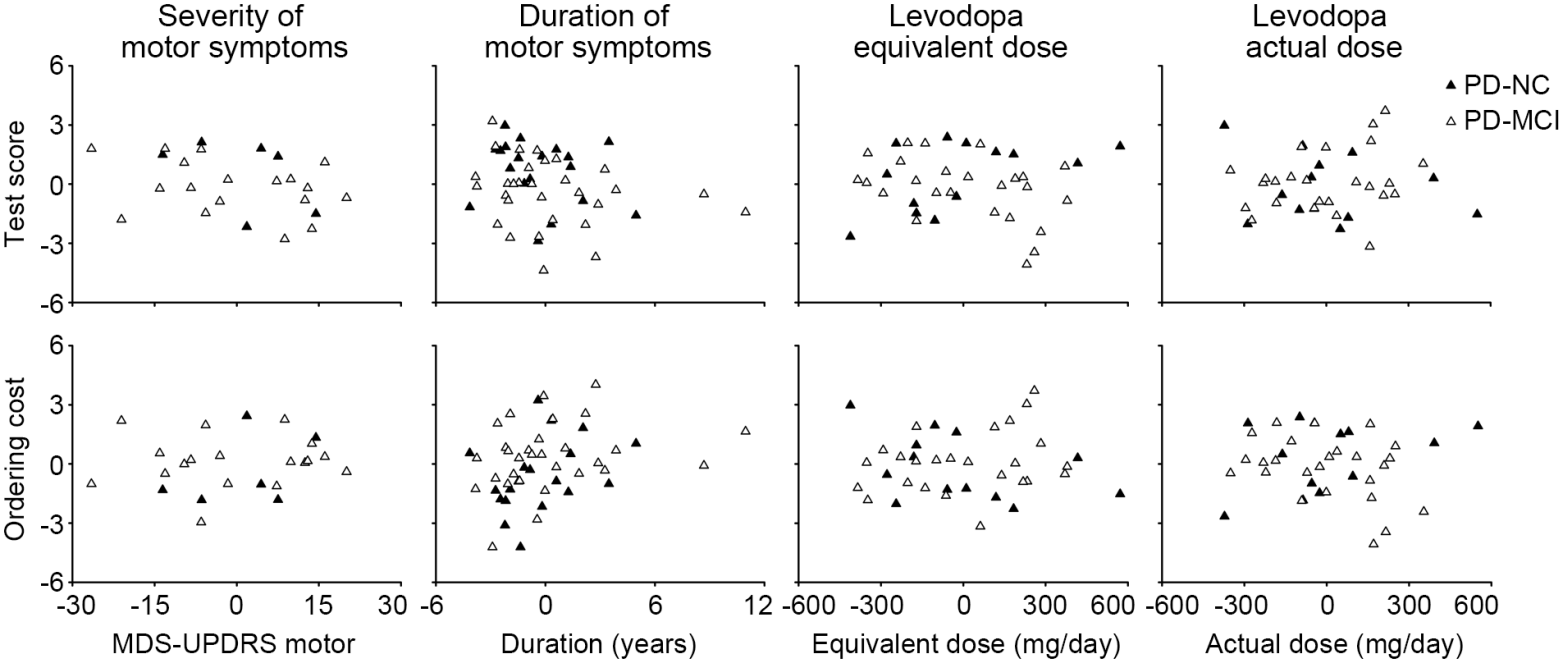
Lack of correlation between individual patients’ task performance and clinical data. Test scores and ordering costs were from the DOT-A. The values were mean-corrected and age-/education-adjusted.

Fig 2 showed the lack of correlation between individual patients’ task performance and their clinical data, using representative data of the DOT-A. Neither test scores nor ordering costs were correlated with the severity of motor symptoms (MDS-UPDRS III motor score), duration of motor symptoms, levodopa equivalent daily dose or actual daily dose, when age and education were controlled. The absence of effect suggested that serial ordering deficits may be unrelated to the dopaminergic dysfunction underlying motor symptoms.

## Discussion

This study investigated serial ordering in nondemented patients with mild PD. We found that the ability to rearrange sequential representations in working memory was already reduced in PD patients with normal global cognition and became worse in those with MCI. Namely, PD patients showed lower test scores and larger ordering costs than healthy controls in both digit ordering and backward tests. In contrast, their ability to encode and maintain the representations in original order was preserved. The ability of serial ordering also declined as a function of age regardless of the group. However, individual patients’ performance (raw score and ordering cost) of either test was unrelated to their severity or duration of motor symptoms, or daily exposure to dopaminergic drugs.

This study confirmed that impaired manipulation and intact maintenance of serial order are characteristic during early stages of PD. This deficit pattern is distinguished from that of patients with Alzheimer’s disease who have problems in both reordering and holding sequences [15]. PD’s ordering difficulties have been observed in previous studies using variable behavioral paradigms (e.g. picture arrangement [6, 7], sentence comprehension [4, 5, 8]) but were often attributed to a general deficit in executive or working memory resources. The nature of the defect was not specified. Here we proposed that it is a selective deficit in the manipulation of serial order and tested the hypothesis by contrasting a digit ordering test with a digit span forward test.

Different from previous studies that pooled PD patients at early and advanced stages [6, 10], this study focused on PD patients with mild motor symptoms (i.e. unilateral or bilateral involvement without balance problems) and screened dementia with a more sensitive instrument (for a comparison of MoCA and MMSE, see [14, 16, 17]). Two earlier studies have examined unmedicated patients’ difficulties in rearranging events or words, but in much smaller samples (only 8–10 patients) [8, 9]. Note, unmedicated patients were not necessarily less severe in motor symptoms (e.g. the patients of [9] had an average UPDRS motor score of 37.3, range from 22 to 55). This study confirmed the existence of serial ordering deficits in early stages of PD, using a larger sample of patients and healthy controls. Moreover, the lack of correlation between individual patients’ task performance and their clinical features suggests that the impairment of serial ordering may develop in parallel with motor symptoms.

Neurochemical mechanisms of serial ordering are still unclear. It is suggested that the prefrontal dopamine system plays an important role in *visuospatial* working memory, with dopamine D1-class receptors involved for online maintenance of representations and D2-class receptors for flexible updating of task-relevant new information and fast switching between multiple tasks or mental sets [18–20]. However there is insufficient evidence for a similar modulatory role of dopamine in *temporal* working memory. In this study, we observed no correlation between individual patients’ ability of serial ordering and their daily exposure to dopaminergic drugs. In an earlier study by Cooper et al. [21], positive effects of levodopa and the dopamine D2 receptor agonist bromocriptine on serial ordering performance only reached borderline significance (also see [22]). Inconsistently, the selective dopamine D2 receptor antagonist sulpiride has been shown to enhance serial ordering performance in healthy adults [23]. In a recent randomized double-blind placebo-controlled multicenter study, Hanagasi et al. reported that a 12-week treatment with selective monoamine oxidase type-B inhibitor rasagiline improved the digit span backward test but not DOT-A [24]. The dopamine hypothesis of serial ordering needs to be tested in further studies with direct pharmacological interventions.

Alternatively it is hypothesized that central acetylcholine or noradrenaline modulates serial ordering. To our best knowledge, only one PD study examined the acetylcholine hypothesis but observed no behavioral change in serial ordering tasks in PD patients who were chronically treated with anticholinergic drugs (e.g. benzhexol, orphenadrine) [21]. Evidence in support of the noradrenaline hypothesis is from studies of propranolol and modafinil in healthy young adults. Propranolol, a lipophilic blocker of β_1_- and β_2_-adrenergic receptors, slowed down the manipulation of serial order [25]. Moreover, modafinil improved the manipulation under difficult ordering rules [26]. Even though there lacks clear consensus about the exact mechanism of action of modafinil, it is recently proposed that modafinil modulates cognition via elevating prefrontal noradrenaline levels and stimulating α_1_- and α_2_-adrenergic receptors [27]. Whether the noradrenaline hypothesis holds in PD patients and how noradrenaline modulates serial ordering are open questions for future research.

This study has methodological limitations. The current categorization of PD-MCI patients was based on an abbreviated cognitive assessment (Level I assessment) rather than comprehensive testing that covers five cognitive domains (i.e. working memory and attention, executive functions, visuospatial functions, language and memory) and includes two tests within each domain (Level II assessment) [13]. It was partly due to the lack of a neuropsychological assessment battery for Chinese PD patients who on average complete fewer years of education than western PD populations. There are difficulties in applying the MDS-recommended neuropsychological tests in Chinese PD patients [28]. For example, Chinese patients are unfamiliar with certain pictures in the Boston naming test such as igloo and unicorn. Due to their motor symptoms, PD patients could hardly conduct the symbol digit modalities test which requires them to manually substitute a sequence of random numbers with corresponding symbols within 90 seconds.

## Conclusions

Temporal working memory is impaired in nondemented patients with mild PD, even in those with normal global cognition. In particular, PD patients’ ability to rearrange sequential representations was reduced, while their ability to encode and maintain original sequences was preserved. The ability of serial ordering also decreased as a function of age regardless of the group. However, PD's reduction of serial ordering ability may not be related to the dopaminergic dysfunction underlying motor symptoms.
